# Effects of social media tourism information quality on destination travel intention: Mediation effect of self-congruity and trust

**DOI:** 10.3389/fpsyg.2022.1049149

**Published:** 2022-12-22

**Authors:** Huimin Wang, Jinzhe Yan

**Affiliations:** ^1^School of Logistics, Transportation and Tourism, Jiangsu Vocational College of Finance and Economics, Huaian, Jiangsu, China; ^2^College of Business, Gachon University, Seongnam, Republic of Korea

**Keywords:** information quality, travel intention, self-congruity, trust, prior knowledge

## Abstract

The asymmetry of tourism information makes social media an important information source. Previous research has been conducted on the influence of tourist-generated content on tourism consumption behavior, but few studies have concentrated on the mechanism of tourism information quality on consumers’ travel intention in the social media environment. Adopting the Elaboration Likelihood Model, this paper aims to investigate how the information quality of social media affects consumers’ travel intention rationally and emotionally and the moderation effect of tourists’ prior knowledge. The empirical results indicate that the quality of social media information positively affects travel intention and self-congruity and trust mediates the relationship between the quality of social media information and travel intention. Moreover, this study identified that tourists’ prior knowledge negatively modifies the relationship between information quality and self-congruity in line with the proposed hypotheses. The research explores the influence mechanism of tourist-generated content quality on consumers’ travel intention, which benefits destination management and content marketing.

## 1 Introduction

Information is the basis of decision-making, and any decision-making is based on the collection, analysis, and evaluation of information, so is the decision-making behavior of tourism consumers. As the pyramid of Data, Information, Knowledge, and Wisdom (DIKW) puts forward, data is the source of information, information is the cornerstone of knowledge, knowledge is the basis and condition of wisdom, and wisdom is the application and productive use of knowledge (Rowley, 2007). On the whole, from data to wisdom, it is a process of continuous processing of data, which is a spiral process and a process of data generating value. With the arrival of the big data era, massive data has penetrated into every industry and field and become an important factor of production. In the Web 2.0 environment, consumers’ travel decision-making behavior relies more on the management and utilization of data and information than ever before. Through social networks, blogs, etc., dormant data on the Web flows in two directions (between users and data providers), making it easier for users to participate in and expand information and transform it into knowledge. However, the availability of information and the creation of knowledge/wisdom do not grow at the same speed (Malik et al., 2018). That makes it more urgent to study how information can be more efficiently transformed into knowledge and wisdom, i.e., how this information is processed through their cognitive perspective to drive the decision-making process.

Social media has greatly improved consumers’ ability to acquire information and knowledge about public events, products, and services (Bertot et al., 2010). In addition, social media improves information exchange, reduces uncertainty, and brings users a sense of belonging (Zehrer and Grabmüller, 2012), fundamentally changing individual travel plans and consumption patterns of travel and leisure (Hudson and Thal, 2013). Therefore, when consumers search for online tourism information, social media has become the essential way (Xiang and Gretzel, 2010) and a prominent place of creating, distributing, and marketing content that is unique to the users (Sin et al., 2020). Even in the middle of the COVID-19 pandemic, the rising UGC content related to tourism impacts numerous consumers to travel (Flores-Ruiz et al., 2021). Hanafiah et al. (2022) documented that social media still plays a vital role in influencing travel intention during the COVID-19 pandemic.

Accordingly, the influence of social media travel information on consumer behavior has become a hot topic in academic research. Some studies focus on adopting social media tourism information and use the technology acceptance model to explain the motivation or influencing factors of potential tourists’ adoption of social media information (Chung et al., 2015; Cheunkamon et al., 2020). It is suggested that potential tourists are more inclined to use the contents of social media with similar interests to themselves when making travel plans (Ayeh et al., 2013). The Elaboration Likelihood Model (ELM) documented that potential tourists’ adoption of online review information is influenced by the dual path factors of the central and peripheral path (Filieri and McLeay, 2014). Some scholars focused on social media tourism information or e-word-of-mouth influencing tourists’ decision-making behaviors (such as hotel and destination choices). Chung and Han (2017) used the ELM model to verify the persuasive effect of social media tourism information on tourism decision-making. Kapoor et al. (2022) proposed that information quality positively impacts hotel stay intention.

Notably, the ELM theory is a classical framework for interpreting the influence of social media tourism information or TGC on tourist behavior. However, the ELM framework only focuses on the direct influence of persuasion factors on consumer behavior without considering the psychological transformation process of potential tourists. Many existing studies use consumers’ attitudes or perceived destination impressions as mediators (Yadav et al., 2021; Kapoor et al., 2022), while few studies proposed psychological factors behind the changes in tourists’ attitudes.

Social media has given consumers a novel experience. Compared with the promotional content of businesses, the emotional evaluation of destinations and their perceived credibility of tourist-generated content (TGC) may play a greater role in consumers’ decision-making process (Iordanova and Stainton, 2019).

Tourist-generated content is not only the explicit content created, published, and shared by users but also includes implicit content, such as user identity, status, relationship, and reputation. This implicit content acts as symbolic clues to stimulate consumers’ association and associate typical tourist images of destinations with their personality characteristics. As a result, the destination image becomes an available resource for self-expression (Elliott and Wattanasuwan, 1998) and an extension of self (Belk, 1988). Morand et al. (2021) also highlight the influence of tourism ambassadors as destination image inducers within the online realm. In other words, in the social media environment, destination symbolism significantly changes tourists’ attitudes. So far, few studies combine the symbolic meaning of destination with the above-mentioned dual-path persuade model.

To compensate for the deficiency, this study proposes an integrated rational and emotional decision path to explain how tourism information on social media affects consumers’ travel intention, focusing on explaining the psychological mechanism behind consumers’ emotional decision path. Furthermore, considering that consumer product knowledge is an important factor influencing information processing ability (Petty et al., 1997), this study also examines the moderating effect of tourists’ prior knowledge in different decision-making paths.

## 2 Literature review and hypothesis development

### 2.1 Elaboration likelihood model

The ELM is one of the most frequently used frameworks in information processing studies and a persuasion model. According to this model, there are two paths to persuade consumers to form and change their attitudes: the core and peripheral paths (Petty et al., 1983). The central path refers to consumers’ comprehensive thinking and analysis of information, forming or changing their attitudes toward products. In contrast to the central path, the peripheral path means that consumers change their attitudes through peripheral clues or implicit hints, which are simple rules or information shortcuts such as brand image and source attractiveness that consumers use to assess a recommendation rather than evaluating the quality of the arguments used by a source (Petty and Cacioppo, 1986).

This study uses social media tourism information quality as an explanatory variable to construct a dual decision-making path model. The core path of ELM corresponds to the rational decision-making path of tourists, which is reflected in that consumers get the perception of destination and generate travel intention through in-depth reasoning and thinking of tourism information itself, that is, the direct influence of tourism information quality on consumers’ travel intention. The peripheral path corresponds to the emotional path, which includes two clues. First, the symbolic meaning of the destination gives consumers the possibility of self-construction. When the self-image of the viewer matches the image of the publisher, the destination becomes a source for individual self-expression, which can easily arouse the viewer’s resonance. The second clue is the trust or emotional experience the tourism destination brings to tourists, especially when consumers cannot form the impression of the destination from the perspective of rational cognition. The feeling or emotional experience brought by tourism information has become the key factor for tourists to make decisions.

### 2.2 Tourism information quality on social media

The immateriality and simultaneity of production and consumption of tourism products determine that tourists usually search for information in order to reduce risks and uncertainties when making travel decisions. Tourists create and share destination tourism information through various social media platforms (blogs and microblogs, content or virtual communities, and social networks) (Tsiakali, 2015) and produce a large number of user-generated content (UGC). Social media is turned into a collection of tourist destination images (Luo and Zhong, 2015), which influence tourists’ cognition and choice of destination (Nezakati et al., 2015). More and more consumers take tourism information on social media as an essential reference when choosing destinations (Chung et al., 2015). In this study, tourism information is defined as “tourist-generated content (TGC) including texts, pictures, and videos about tourist destinations on social media platforms.” TGC or the shared memorable tourism experiences are both cognitive and emotional (Kim et al., 2012), and are inseparable from tourists’ behavioral engagements (Servidio and Ruffolo, 2016). In other words, the more tourists participate in the activities, the better they can retrieve the memories (Coudounaris and Sthapit, 2017) and present them on social platforms. Furthermore, the distinctiveness of tourists’ memorable tourism experiences is crucial for destination management and marketing (Wearing and Foley, 2017). By classifying city attractions, Yu et al. (2021) proposed to identify the unique patterns of attractions to recognize what can be a memorable cue or stimuli of tourists’ shared memorable experiences on social media.

As Yeap et al. (2014) stated, information quality is “how the provided information is useful for the consumer.” Information quality is a strong predictor of the credibility of information sources and website quality (Filieri et al., 2015), indicating that the quality of information content itself is the core factor in persuading consumers. Our study follows this argument and takes information quality as the independent variable. Different scholars put forward their own opinions on the measurement of information quality. For example, based on the characteristics of the information content itself, the quality of content can be measured from the four indicators of relevance, understandability, adequacy, and objectivity (Park et al., 2007), or authenticity, authority, and relevance (Wang, 1998), value (Filieri and McLeay, 2014), accuracy and completeness (Zhang et al., 2014), richness and usefulness (Bovee, 2004).

The marketing value of information quality is that it has a significant impact on consumers’ willingness to adopt information and purchase decisions. The quality and characteristics of online information will affect tourists’ decision-making. For example, information accuracy, relevance, and timeliness will affect tourists’ adoption behavior of online comment information (Filieri and McLeay, 2014). Positive UGC can stimulate consumers to produce both emotional (motivation and pleasure) and cognitive responses (perceived information quality), form direct behavioral responses (information sharing and direct purchase), and potential behavioral responses (future purchase intention and brand commitment), respectively (Kim and Johnson, 2016).

### 2.3 Travel intention

Behavioral intention usually refers to an individual’s possibility or attitude tendency to take action on an activity or object (Smith, 2004). Purchase intention is also considered the most effective predictor of consumer purchase behavior (Morwitz and Schmittlein, 1992). The intention is used to predict various consumer behaviors, including travel decision-making behaviors, and researchers can learn how individuals will act from their behavioral intentions (Sheeran, 2002). Travel intention is the main driving force for tourists to travel to destinations (Woodside and Lysonski, 1989). It can predict tourists’ travel behavior and is the tendency of individuals’ expectations, plans, or intentions on whether their future behavior will be carried out (Lam and Hsu, 2006).

Consumers’ impression of products or services is formed through processing various information sources. If the information content is perceived to be complete, accurate, relevant, and authentic, it is easy for consumers to pay attention to and deeply process it and form a rational cognition and attitude toward the destination. The higher the quality of online reviews, the stronger the purchase intention of consumers (Park et al., 2007). High-quality information enables users to understand specific products or services better, gain support, and be able to make better decisions (Kim et al., 2017). Many scholars have empirically tested that online travel reviews significantly positively impact consumers’ booking intentions (Lu et al., 2013; Sparks et al., 2013; Torres et al., 2015; Zhao et al., 2015). Lata and Rana (2021) also verified that information quality is a predictor for online hotel booking intentions. Based on the above literature, this study presumes H1.

H1 Social media tourism information quality positively impacts consumers’ travel intention.

### 2.4 Self-congruity

Self-congruity stems from one of the core constructs of social psychology: self-concept. Self-concept can be understood as self-image. It is an individual’s comprehensive evaluation of his own behavior, ability, values, and other aspects. It is a subjective perception and cannot be directly observed. “Protecting, maintaining, and promoting one’s self-concept or symbolic self is one of the most basic goals of human behavior” (Onkvisit and Shaw, 1987). Self-congruity is an extension of self-concept, also known as self-concept congruity or self-image congruity. Sirgy et al. (1991) proposed that self-congruity refers to “the degree of matching or consistency between the symbolic image of a product/brand and the self-image of customers.” In tourism, self-congruity refers to matching tourists’ self-image and typical image.

The degree of consistency between consumers’ self-concept and product user image will affect consumers’ attitudes toward products (Sirgy, 1982). The symbolic meaning of a brand can explain this: all social behaviors have symbolic meaning, and consumers can show their public image and construct their desired identity by using a brand (Helgeson and Supphellen, 2004). Symbolic consumption stems from socialized human behavior–human beings constantly construct, maintain, promote, transform, and express their “self” in social behavior (Elliott and Wattanasuwan, 1998; Escalas and Bettman, 2005). Therefore, consumption is a universal behavior of human beings, and brands become a resource for people to obtain symbolic meaning in the consumption process. Consuming a certain brand and being associated with a brand image becomes a means for consumers to construct, transform, and express themselves in daily life (McCracken, 1987). Thus, the symbolic meaning of a brand is actually a projection of consumers’ self-concept of the brand. For instance, the perceived luxuriousness of a coffee shop leads to high self-congruity, and thus increasing customers’ willingness to pay a price premium (Li et al., 2022).

Like brand images, destination images are also symbolic. Chon (1992) was the first to introduce self-congruity into the field of tourism, and he found that the higher the degree of self-congruity of tourists is, the more satisfied they are with the destination. People identify with brands or businesses that help define or reinforce, improve or enhance, and communicate their self-concept to others or society. This identification significantly impacts attitudes and behaviors such as purchasing intention, recommendation intention, price sensitivity, and loyalty (Bhattacharya and Sen, 2003). Ahn et al. (2013) proposed that self-congruity affects tourists’ destination choice behavior. Egota et al. (2022) verified that self-congruity directly impacts destination satisfaction, engagement, and expectations.

Consumers are more attracted to the information posted by like-minded publishers. It is obvious that when consumers read other users’ reviews or content, they look for similarities with their preferences and profiles (Tsiakali, 2015). Users on the Internet are more likely to collect information that supports their worldview, exclude different information, and build polarized communities around shared narratives (Yu and Ko, 2021). Furthermore, viewers always process self-related information and then deal with unrelated information (Fast and Tiedens, 2010). This is because the highly relevant information is easier to notice and recognize, helping maintain a consistent self-image. Based on the literature, the following hypotheses are driven:

H2 The quality of social media tourism information positively affects self-congruity.

H3 Self-congruity plays a mediating role in the effect of information quality on travel intention.

### 2.5 Trust

Trust is the confident and positive expectation of an individual to another individual or organization in social communication under the circumstance of risk (Moorman et al., 1992). Mayer et al. (1995) proposed that trust is composed of the trustor’s perception of the trustee’s competence, benevolence, and integrity, which indicates the willingness of the individual to bear risks in the transaction and reflects the individual’s cognition of the transaction risks. In the field of tourism, trust is a kind of confidence, belief, and expectation that consumers hold in the tourism destination, and they are willing to believe that the tourism destination has the ability and can meet the needs of consumers in tourism as promised.

Because of the asymmetry of tourism information, consumers cannot experience the quality of tourism products before arriving at the destination. In order to obtain more accurate destination perception, consumers tend to obtain information through more reliable channels. The content shared by users of social media is mostly from consumers’ own experiences rather than business publicity. Due to its non-trading attribute and open access, UGC is regarded as more objective and fair (Ridings et al., 2002), which provides important decision-making reference for consumers to search for tourism information. Compared with promotional materials provided by tourist boards and commercial enterprises, the credibility of UGC is higher, and the perceived credibility of the destination may play a greater role in the consumer decision-making process (Iordanova and Stainton, 2019). Although travel-related UGC is more reliable than information created or uploaded by official tourism organizations (Fotis et al., 2012), there are cases where the user is concerned about their trust in the reliability of online travel reviews as the sources can modify and misuse in various ways (Fan et al., 2018). Sparks et al. (2013) found that user-generated and detailed information is an important clue to trust. Mahat and Hanafiah (2020) documented that information’s accuracy, reliability, confidentiality, and privacy lead consumers to trust information sources. In the social media environment, trust significantly impacts purchase intention (Hajli, 2014). Trust is vital for online tourism marketing because it increases the interest in purchase behavior (Li et al., 2020). Based on the literature, the following hypotheses are driven:

H4 The quality of social media tourism information positively affects trust.

H5 Trust plays a mediating role in the effect of information quality on destination tourism intention.

Tourists have a high degree of trust in destinations because of their similar characteristics, and also have a high sense of identity with destinations that help define, strengthen, and improve their self-concept and reduce the inconsistency between ideal and reality. Self-image consistency will promote consumers’ attachment to the product, induce consumers’ emotional commitment to the brand, improve the relationship between consumers and the brand, and show a kind of emotional trust (Kressmann et al., 2006). The higher the consistency between consumers and information publishers is, the higher the trust of consumers in users’ published content, which shows that self-congruity is the clue of trust (Ayeh et al., 2013). Thus, the hypothesis is proposed:

H6 Trust is mediating in the relationship between self-congruity and travel intention.

### 2.6 Prior knowledge

Prior knowledge is also known as consumer knowledge or consumer expertise, which refers to the relevant knowledge and experience consumers can rely on when choosing products to solve specific consumption problems (Mitchell and Dacin, 1996). Consumer knowledge is divided into familiarity and expertise (Jacoby et al., 1986). As per knowledge hierarchy (DIKW), knowledge is derived from information but is not a subset of information. It is the information that is “understood,” associated with specific situations, and can guide “how” actions. The knowledge can be available in different formats, but analyzing, understanding, and categorizing it requires extra attention to convert it to wisdom (Malik et al., 2018). In the era of information explosion, knowledge eliminates the false and preserves the true, eliminating the coarse and preserving the fine. Knowledge makes information useful and can solve the “how to” problem for a specific recipient in a specific environment, improving the efficiency and quality of work. At the same time, the accumulation and application of knowledge play a very important role in enlightening wisdom and leading the future.

The level of consumers’ knowledge affects how they collect and use information, ultimately affecting their evaluation, purchase, and use of products (Cordell, 1997). As an embodiment of cognitive ability, the knowledge level greatly influences information processing and decision-making (Alba and Hutchinson, 2000).

According to the ELM theory, individual attitude change has central and peripheral pathways, and the ability to process information affects individuals to adopt central or peripheral pathways (Petty et al., 1983). Consumer knowledge is an important factor influencing information processing ability (Petty et al., 1997). Tourists with a high level of prior knowledge have adequate processing and utilization of information, and a more accurate understanding of the meaning of information. They are more inclined to choose the central approach for fine processing and in-depth analysis of information, forming or changing their attitudes to things based on evaluating the quality of the information itself. While tourists with low prior knowledge are less capable of thinking about a message, they are inclined to use peripheral cues (such as emotional stimulation, preference for information expression methods, etc.) to evaluate a message (Petty et al., 1997).

It has been proved that consumers’ prior knowledge is a very important moderating variable in their information processing (Roehm and Sternthal, 2001). Tourists with higher prior knowledge have stronger cognitive needs and are more willing to obtain information before making travel decisions (Teichmann, 2011). Furthermore, consumers with more prior knowledge tend to adopt rational analysis and seldom evaluate products by peripheral cues (Ratchford, 2001), while novice tourists are more inclined to make use of peripheral information or relatively simple clues (such as tourist images of destination and emotional stimulus) for information evaluation (Sirgy and Su, 2000; Beerli et al., 2007). Kumi and Limayem (2012) advocated that high-expertise consumers are more likely to rely on perceived content quality to make the decision, while individuals with low expertise are more likely to rely on contextual factors. That is, tourists’ prior knowledge can strengthen the possibility of tourists’ rational information processing and weaken the possibility of tourists’ emotional information processing. Therefore, the following assumptions are put forward:

H7 Tourists’ prior knowledge positively regulates the impact of tourism information quality on tourism intention.

H8 Tourists’ prior knowledge negatively regulates the impact of information quality on self-congruity.

H9 Tourists’ prior knowledge negatively regulates the impact of information quality on trust.

**FIGURE 1:**
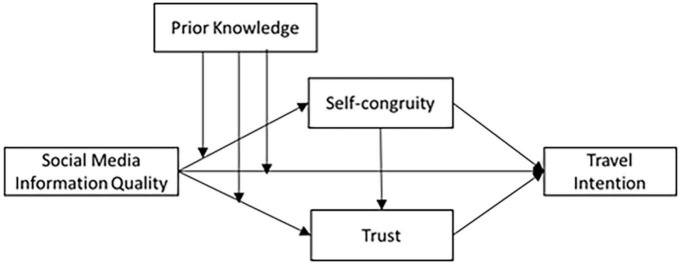
Research model.

Based on the above analysis, the conceptual model (Figure 1) is as follows.

## 3 Research method

### 3.1 Data collection

In order to enhance the reliability of the research results, this study released and collected questionnaires on Credamo.^1^ Credamo platform is a professional survey platform with samples and a strict credit investigation system, and it has provided scientific research and education data services for teachers and students in more than 2,000 colleges and universities around the world, including MIT, New York University, Hong Kong University of science and technology, Peking University, Tsinghua University. The questionnaire was released and collected in March 2022, and each sample was paid 3 Chinese Yuan (CNY). To ensure the accuracy and quality of the results, respondents need to meet two conditions: they are social media users and have browsed TGC. Therefore, at the beginning of the survey, we gave the concept of TGC and corresponding examples and asked respondents to recall their recent experience of browsing the tourism destination information on social media. As a result, a total of 530 samples were collected, and 399 valid ones were left after excluding invalid questionnaires.

Among the valid samples, the proportion of men and women is relatively balanced, accounting for 42.9% (men) and 57.1% (women), respectively. The age group of 29–39 accounts for the largest (54.6%), followed by the age group of 18–28 (36.1%), which is in line with the younger characteristics of social media users. More than 89% of the respondents have a bachelor’s degree or above. They have a good understanding and decision-making ability to ensure data accuracy. Those with incomes between 5,001–15,000 yuan accounted for 64.7 percent of the total, which is consistent with the income level of Chinese residents. In short, from the perspective of demographic characteristics, the sample is well-representative (see Table 1).

**TABLE 1:** Demographic characteristics (*n* = 399).

Dimension	Items	Frequency (*n*)	Percentage (%)
Gender	Male	171	42.9
	Female	228	57.1
Age	18–28 years	144	36.1
	29–39 years	218	54.6
	40–50 years	27	6.8
	51 and elder	10	2.5
Education	High school and below	7	1.8
	College	34	8.5
	University	306	76.7
	Postgraduate and above	52	13
Monthly income	≤5,000 CNY	95	23.8
	5,001–8,000 CNY	171	42.9
	8,001–15,000 CNY	87	21.8
	≥15,001 CNY	46	11.5

### 3.2 Measurement design

To ensure content validity, we use relatively mature scales when measuring variables and make necessary adjustments according to the context of the study. Likert 5 scoring method was adopted (1 = “strongly disagree” and 5 = “strongly agree”). The questionnaire mainly includes three parts: (1) tourists’ preference for social media, (2) key variables, and (3) demographic characteristics. An eight-item scale (Wang, 1998; Bovee, 2004; Park et al., 2007; Zhang et al., 2014) operationalized information quality. Self-congruity mainly refers to Sirgy et al. (1997), including four items; Four items (McAllister, 1995) which were utilized to measure trust. A four-item scale (Kerstetter and Cho, 2004; Füller et al., 2008) was used to measure prior knowledge. Finally, four items (Smith, 2004) were used to assess travel intentions (see Table 2).

**TABLE 2:** Construct reliability and convergent validity.

Variable	Items	References	Factor loading	Alpha	AVE	CR
Information quality (IQ)	The tourist-generated content source is authoritative.	Wang (1998), Bovee (2004), Park et al. (2007), Zhang et al. (2014),	0.852	0.805	0.519	0.810
	The tourist-generated content is reliable. The tourist-generated content is relevant.		0.637 0.687			
	The tourist-generated content is rich.		0.686			
Self-congruity (SC)	The image of a typical tour of the destination is consistent with mine.	Sirgy et al. (1997)	0.783	0.774	0.543	0.781
	People with a similar image to mine tend to go to the destination.		0.688			
	The destination image is consistent with my image.		0.737			
Trust (TRU)	I can rely on this destination.	McAllister (1995)	0.658	0.754	0.521	0.765
	I would feel a sense of loss if I could not visit the place. This is a responsible destination.		0.743			
			0.761			
Prior knowledge (PK)	I have a good knowledge of the destination.	Kerstetter and Cho (2004), Füller et al. (2008)	0.689	0.881	0.656	0.883
	I am familiar with the destination.		0.831			
	Compared to others, I know the destination better.		0.857			
	People around me think I am familiar with the destination.		0.851			
Travel intention (TI)	After browsing tourist-generated content, I have a great possibility of traveling to the destination.	Smith (2004)	0.744	0.765	0.532	0.773
	I am willing to pay more money to go to the destination.		0.773			
	I am willing to recommend the destination to others.		0.667			

### 3.3 Reliability and validity

The reliability of each construct was measured with Cronbach’s Alpha coefficient. As shown in Table 2, the values of all factors were above the recommended threshold of 0.7 based on George and Mallery’s (2003) criterion. The reference scales in this study were all previous mature scales, and confirmatory factor analysis (CFA) was used to test the measurement model. Factor loadings exceeding the recommended 0.5 (Hair et al., 2013) were accepted. The measurement model indices were all within recommended thresholds (*x*^2^/df = 1.385, normed fit index (NFI) = 0.947, goodness of fit index (GFI) = 0.958, standardized root mean squared residual (SRMR) = 0.035, and root mean square error of approximation (RMSEA) = 0.031), indicating that the measurement model achieved acceptable fit (Browne and Cudeck, 1992). To ensure construct validity, we CFA analysis followed by a calculation of average variance (AVE) and composite reliability (CR) to assess the convergent validity of the measurement model. The validity results showed that the average variance (AVE) values exceeded 0.5, and CR values greater than the threshold of 0.7 (see Table 2) recommended by Fornell and Larcker (1981).

Furthermore, we evaluate discriminant validity using the square root of AVE. As shown in Table 3, the square root of the AVE values of each construct was greater than the correlations between pairs of latent variables, indicating that the discriminant validity was satisfactory.

**TABLE 3:** Correlation and discriminant validity.

	1	2	3	4	5
1. Information quality (IQ)	**0.720**				
2. Prior knowledge (PK)	0.373***	**0.810**			
3. Self-congruity (SC)	0.545***	0.445***	**0.737**		
4. Trust (TRU)	0.356***	0.269***	0.339***	**0.722**	
5. Travel intention (TI)	0.531***	0.354***	0.578***	0.523***	**0.729**

*p < 0.05, **p < 0.01, ***p < 0.001.

The values on the diagonal represent the square root of the AVE.

## 4 Hypothesis testing

### 4.1 Main effect test

Firstly, regression analysis was performed to test the effect of tourism information quality on travel intention. The analysis results (Table 4) show that the quality of social media tourism information has a significant positive impact on tourism intention (*b* = 0.372, *p*-value < 0.001), indicating that the main effect is significant supporting H1.

**TABLE 4:** Main effect and mediating effect.

Variable		TI	SC	TRU	TI
Control variables	Gender	Included			
	Age				
	Education				
	Income				
Independent variable	IQ	0.372***	0.526***	0.150***	0.211***
Mediating variable	SC			0.083**	0.198***
	TRU				0.296***
*R* ^2^		0.242	0.231	0.157	0.369
*F*		7.445***	23.657***	12.206***	32.664***

*p < 0.05, **p < 0.01, ***p < 0.001.

### 4.2 Mediating effect test

The test results (Table 4) show that information quality has a significant positive impact on self-congruity (*b* = 0.526, *p*-value < 0.001), and self-congruity plays a positive role in affecting travel intention (*b* = 0.198, *p*-value < 0.001) thus the data supports H2 and H3. Similarly, information quality has a significant positive impact on trust (*b* = 0.150, *p*-value < 0.001), and trust plays a positive role in affecting travel intention (*b* = 0.296, *p*-value < 0.001), supporting H4 and H5. Furthermore, the impact of tourism information quality on tourism intention is still significant (*b* = 0.211, *p*-value < 0.001), indicating that self-congruity and trust partially mediate between information quality and travel intention. Moreover, self-congruity had a significant positive effect on trust (*b* = 0.083, *p*-value < 0.01), both self-congruity (*b* = 0.198, *p*-value < 0.001), and trust (*b* = 0.296, *p*-value < 0.001) had significant positive influences on travel intention, indicating that trust acts as a partial intermediary between self-congruity and travel intention supporting H6. In addition, this study examined the mediating effect size using the bootstrap method (Hayes and Rockwood, 2017). According to the result in Table 5, the bootstrap 95% confidence interval (CI) did not contain 0, indicating the mediating effect was significant, and the total indirect effect accounts for nearly half of the total effect (43.5%).

**TABLE 5:** Mediating effect test.

Mediating path	Estimation	Boot SE	LLCI	ULCI	Percentage
Total indirect effect	0.162	0.033	0.102	0.232	43.5%
Information quality → self-congruity → travel intention	0.104	0.029	0.055	0.169	28.0%
Information quality → trust → travel intention	0.044	0.018	0.014	0.085	11.8%
Information quality → self-congruity → trust→ travel intention	0.013	0.008	0.0003	0.031	3.6%

### 4.3 Moderating effect test

**FIGURE 2:**
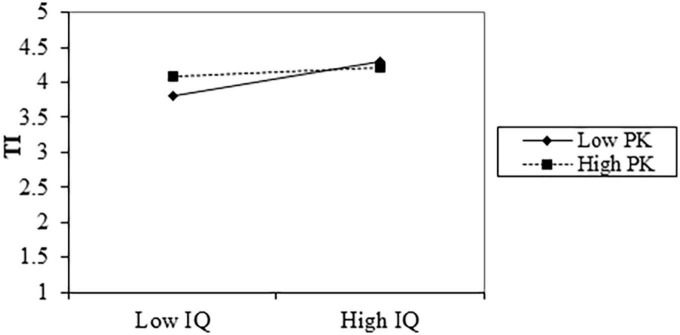
Interaction of information quality (IQ) and prior knowledge (PK) on travel intention (TI).

A series of regression analyses using PROCESS (Hayes and Rockwood, 2017) was conducted to test the moderating effect further. We test the moderating effect of tourists’ prior knowledge on the relationship between information quality and tourism intention. The results in Table 6 show that the interaction term has a significant negative impact (β = −0.234, *p*-value < 0.001) on tourism intention rather than a positive role mentioned in Hypothesis 7. Next, spotlight analysis was used to test the moderating effect further. Based on one standard deviation of the average value of tourists’ prior knowledge, tourists were divided into tourists with high (M + 1SD) prior knowledge (PK) and low (M − 1SD) PK for simple slope analysis (Figure 2). The results show that compared with tourists with low PK (Simple Slope = 0.569, *p*-value < 0.001), tourists with high PK (Simple Slope = 0.135, *p*-value < 0.05) are less possibly influenced by tourism information on the social media platform, which means that the more knowledge they have about destinations, the less they rely on information when making travel decisions.

**TABLE 6:** Moderating effect test.

Variable		TI	SC	TRU	TI
Control variables	Gender	Included			
	Age				
	Education				
	Income				
Independent variable	IQ	0.352***	0.453***	0.178***	0.222***
Moderating variable	PK	0.055*	0.156***	0.034	0.018
Mediating variable	SC				0.176***
	TRU				0.285***
Interaction	IQ × PK	−0.234***	−0.176*	–0.052	−0.189***
*R* ^2^		0.295	0.288	0.151	0.397
*F*		23.399***	22.598***	9.962***	28.458***

*p < 0.05, **p < 0.01, ***p < 0.001.

**FIGURE 3:**
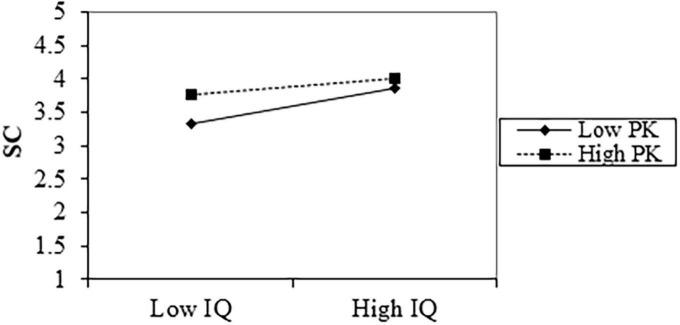
Interaction of information quality (IQ) and prior knowledge (PK) on self-congruity (SC).

Then a moderated mediation model (Hayes and Rockwood, 2017) was conducted to test whether the mediating effect would be weakened or strengthened when the level of moderating variable was changed. Table 6 shows that the interaction of information quality and prior knowledge has a negative effect on self-congruity (*b* = −0.176, *p*-value < 0.05). The interaction effects at different levels of prior knowledge (the mean of prior knowledge ± 1 SD) were further examined, and it turned out that the relationship between information quality and self-congruity is stronger when prior knowledge is low (*b* = 0.615, *p* < 0.001) rather than high (*b* = 0.290, *p*-value < 0.01; Figure 3). Table 7 shows the conditional indirect effect of information quality on travel intention through self-congruity at different levels of the moderating variable prior knowledge (M ± 1SD). The indirect effect was strong for lower PK group [β = 0.108, 95% CI: (0.051, 0.185)] and was weak for higher PK group [*b* = 0.051, 95% CI: (0.017, 0.110)]. Thus, Hypothesis 8 was supported. As the interaction items of information quality and prior knowledge did not significantly influence trust (*b* = −0.052, *p*-value > 0.05), rejecting H9.

**TABLE 7:** Conditional indirect effects of information quality (IQ) on travel intention (TI) *via* self-congruity (SC) at levels of prior knowledge (PK).

Mediating variable	Grouping of moderating	Effect value	Boot SE	Boot LLCI	Boot ULCI
SC	Low PK	0.108	0.034	0.051	0.185
	High PK	0.051	0.024	0.017	0.11

In addition, we examined the conditional direct effect of information quality on travel intention after adding two mediator variables. Overall, the interaction of information quality and prior knowledge has a significant negative effect (*b* = −0.176, *p*-value < 0.05) on travel intention (Table 6), while this effect is mainly reflected in the group with low prior knowledge (*b* = 0.397, *p*-value < 0.001). However, for tourists with a high prior knowledge level (M + 1SD), the impact of tourism information quality on travel intention is no longer significant (*b* = 0.047, *p*-value > 0.05).

## 5 Discussion, implications, and limitations and future work

### 5.1 Discussion

Integrating rational and emotional perspectives, this study explores tourism information quality’s direct and indirect impacts on consumers’ travel intentions. In addition, the moderating role of prior knowledge in the influence of information quality on rational and emotional decision-making paths is tested.

The empirical results show that consumers’ processing of tourism information and making tourism decisions are the results of both rationality and sensibility, and self-congruity and trust play a mediating role in emotional decision-making. The results align with Levy’s (1959) assertion that consumers are not functionally oriented and the symbolic meaning of products largely influences their behavior. It also proves that, as Sirgy and Su (2000) proposed, the more consistent tourists’ self-concept with the image of the destination, the more positive they will be toward the destination and have the intention to visit.

It is found that tourists’ prior knowledge negatively moderates the direct impact of social media tourism information quality on tourism intention. Compared with expert tourists, novice tourists’ tourism decisions are more dependent on tourism information content. It is contrary to the view proposed by Teichmann (2011) that the higher the level of professional knowledge of tourists, the more inclined they are to obtain information from outside before making tourism decisions. The possible reason is that consumers’ familiarity with the destination is inversely related to their willingness to use the platform (Abd Aziz et al., 2010). Tourists with a high level of prior knowledge have rich destination knowledge or personal experience, so they can better identify the information about the destination on the social media platform, and their attitude toward the destination no longer depends too much on the information contained on the media platform.

The analysis results also show that factors such as travel knowledge and previous travel experience have a negative moderating effect on the relationship between information quality and self-congruity, consistent with Beerli et al. (2007). Novice travelers rely more on relatively simple information cues, such as the image of a destination’s visitors. The information shared by typical tourists is transformed into a symbolic and emotional information clue, which stimulates the novice tourists to carry out the association, draw their own image close to the image of the destination, and invest emotional commitment to the destination so as to achieve “balance” in the way of emotional decision-making.

It must be pointed out that in the Web 2.0 era, social media was not only an information platform but also an influence platform (Hanna et al., 2011), enabling consumers to have more power than ever before. When interests are damaged, consumers can express their dissatisfaction through negative word of mouth, which can have great negative impacts on the destination image as well as the sustainable development of the local tourism industry (Liu et al., 2020). Thus, all stakeholders should work toward transforming the tourism market from unregulated to regulated (Liu et al., 2021).

### 5.2 Implications

#### 5.2.1 Theoretical implications

First, this study innovatively integrates rational and emotional decision-making paths to explore the impact of social media tourism information on consumers’ tourism intention. The generation of tourism intention comes not only from the rational evaluation based on functional information clues but also from the emotional evaluation based on self-congruity and trust. It is verified that the tourist destination has both functional value and symbolic value for tourists. Second, from the perspective of self-congruity, this study explores the behavior tendency before traveling. Previous studies mainly focused on the impact of self-congruity on post-tour behavior, such as tourist satisfaction (Murphy et al., 2007) and revisit intention (Matzler et al., 2016). This study enriched the research on the impact of self-congruity on consumers’ pre-tour behavior and confirmed that self-congruity also impacts consumers’ destination behavior intention before traveling. Third, it verifies the boundary conditions of the tourism information quality on tourism decision-making. Information asymmetry is the premise of the value of social media. Because of the asymmetry of tourism information, consumers pay more attention to the non-trading attribute of social media and tend to offset uncertainty perception with the help of user-shared information. When the asymmetry of tourism information decreases, the impact of social media tourism information will also decline, and tourists’ prior knowledge can offset tourists’ dependence on tourism information. Expert tourists are more insensitive to the risk of tourism information asymmetry than novice tourists. As the empirical analysis results of this study show, the impact of tourism information quality on tourism intention is more obvious among novice tourists.

#### 5.2.2 Practical implications

Social media has endowed tourism consumers with more ways of self-expression and value demands. In the context of “attention economy,” this study provides empirical support for guiding destination operators to carry out content marketing, destination image building, and tourism enterprise service innovation. First, this study found that the quality of TGC directly affects consumers’ travel intentions and indirectly affects consumers’ travel intentions through self-congruity and trust. Therefore, tourism destination management should pay attention to the incentive and management of TGC, and encourage tourists to create value together. On the one hand, tourism managers should encourage publishers to continue to create and share high-quality tourism information that is complete, rich, eye-catching, authentic, and credible so that visitors, especially consumers who have not visited the destination, can form a clearer and rational understanding of the destination through the functional attributes of information transmission. On the other hand, the social attributes of media platforms should be utilized to create opinion leaders and destination spokespeople through hidden attributes such as identity, relationship, and popularity of publishers, to stimulate consumers’ emotional experience and value demands for maintaining their own image. For consumers are not only “motivated by reason” but also “motivated by emotion.” Especially, the destination administrations should establish a good image and conduct transparent supervision on the unethical incident timely and efficiently. Second, this study found that the more consistent the consumer’s self-image with the destination image, the easier it is to generate tourism intention. Therefore, the tourism destination management party should not only pay attention to the dissemination of functional information but also use the symbolic meaning of the destination to design publicity information to shape their personality and differentiation advantages. It will stimulate consumers to regard the destination image as an extension of their self-image, so that consumers can express their personality and maintain their image by traveling to the destination. Third, the direct impact of the quality of TGC on tourism intention mainly exists in the novice tourist group, while the role of the expert tourist group can be almost ignored. Therefore, for novice tourists, tourism destination operators or managers should pay attention to guiding and encouraging publishers to introduce the security factors such as tourism destination services and infrastructure that novice tourists are concerned about so as to weaken the negative impact of cognitive bias on tourism intention. Especially during the pandemic, fear of COVID-19 and perceived risk significantly negatively impact attitude (Bratić et al., 2021; Rather, 2021). Destination operators should use social platforms to pass on authentic information about safety measures to visitors to minimize tourist’s negative feelings and diminish the perceived fear of COVID-19.

This study also found that novice tourists are more likely to rely on symbolic cues or emotional cues of tourism information than expert tourists. Therefore, the choice of publishers is very important. When the image of publishers is consistent with the brand image, it is easier for visitors to remember the brand (Knoll et al., 2015). Publishers with a higher matching degree with the brand/destination image are more likely to arouse positive interaction of visitors and lead to a positive attitude toward the destination.

In conclusion, this study provides a new research perspective on how social media travel information affects the decision-making of potential travel consumers. Furthermore, it verifies the symbolic value of a tourism destination and provides a theoretical reference for optimizing its brand image value. At the same time, it provides support for destination management to carry out content marketing.

### 5.3 Limitation and future work

The world tourism industry has been inevitably influenced a lot due to unprecedented mobility restrictions caused by the novel coronavirus (COVID-19) (Gössling et al., 2020). Nevertheless, we did not consider the epidemic’s influence as a leading factor because of the following considerations. First, it is often difficult to reasonably operate the independent variable of COVID-19 when collecting data from the questionnaire. Future research should encourage the application of big data analysis techniques (including natural language processing, machine learning, etc.) and experimental methods to gain more and deeper insights into changes in tourist behavior and new tourism consumption phenomena brought about by the COVID-19 pandemic (Chen and Li, 2022). Besides, China may be different from other countries and regions in terms of epidemic prevention policies. How to localize epidemy-related tourist behavior studies in China is also a topic that need attention and future directions.

In addition to the above, this study still has several limitations that lead to directions for future work. First, this study took the tourism information quality on social media as an overall dimension to test its impact mechanism on consumers’ travel intention and did not subdivide it into functional and emotional information cues, which can be further tested in future work. Second, we use prior knowledge as the moderating variable in this study. Finally, since consumer involvement (Geng and Chen, 2021; Yadav et al., 2021) may influence consumer decision-making, involvement can be tested as a moderating variable in the future. Third, the empirical data of this study only covers vacation tourism destinations in China, and whether the findings are applied to other destination types should be further verified.

## Data availability statement

The raw data supporting the conclusions of this article will be made available by the authors, without undue reservation.

## Author contributions

HW: formal analysis, writing—original draft preparation, and supervision. JY: visualization and project administration. Both authors contributed to the conceptualization, methodology, validation, data curation, writing—review and editing, and read and agreed to the published version of the manuscript.

## Footnotes

1
https://www.credamo.com
